# Global health security and universal health coverage: from a marriage of convenience to a strategic, effective partnership

**DOI:** 10.1136/bmjgh-2018-001145

**Published:** 2019-01-13

**Authors:** Clare Wenham, Rebecca Katz, Charles Birungi, Lisa Boden, Mark Eccleston-Turner, Lawrence Gostin, Renzo Guinto, Mark Hellowell, Kristine Husøy Onarheim, Joshua Hutton, Anuj Kapilashrami, Emily Mendenhall, Alexandra Phelan, Marlee Tichenor, Devi Sridhar

**Affiliations:** 1 Department of Health Policy, London School of Economics and Political Science, London, UK; 2 Center for Global Health Science and Security, Georgetown University, Washington, District of Columbia, USA; 3 Institute for Global Health, University College London, London, UK; 4 UNAIDS, Geneva, Switzerland; 5 Global Academy of Agriculture and FoodSecurity, The Royal (Dick) School of Veterinary Studies and The Roslin Institute, University of Edinburgh, Edinburgh, UK; 6 Keele University, Keele, UK; 7 O'Neill Institute for National and Global Health Law, Georgetown University Law Centre, Georgetown University, Washington, District of Columbia, USA; 8 Harvard University T H Chan School of Public Health, Boston, Massachusetts, USA; 9 Global Health Policy Unit, University of Edinburgh, Edinburgh, United Kingdom; 10 Department of Global Public Health and Primary Care, University of Bergen, Bergen, Norway; 11 University of Sussex, Brighton, UK; 12 Centre for Global Public Health, Queen Mary University, London, London, UK; 13 Georgetown University Edmund A Walsh School of Foreign Service, Washington, District of Columbia, USA; 14 Georgetown University O'Neill Institute for National and Global Health Law, Washington, District of Columbia, USA; 15 University of Edinburgh Division of Health Sciences, Edinburgh, UK

**Keywords:** universal health coverage, global health security, health systems strengthening, risk, human rights

## Abstract

Global health security and universal health coverage have been frequently considered as “two sides of the same coin”. Yet, greater analysis is required as to whether and where these two ideals converge, and what important differences exist. A consequence of ignoring their individual characteristics is to distort global and local health priorities in an effort to streamline policymaking and funding activities. This paper examines the areas of convergence and divergence between global health security and universal health coverage, both conceptually and empirically. We consider analytical concepts of risk and human rights as fundamental to both goals, but also identify differences in priorities between the two ideals. We support the argument that the process of health system strengthening provides the most promising mechanism of benefiting both goals.

**Summary box**

**What is already known about this subject?**

- Universal health coverage (UHC) and global health security (GHS) are frequently being used in tandem by policymakers, recognising that there are synergies between the two parallel agendas.

**What are the new findings?**

- UHC and GHS goals are in tension. The research and practice communities that represent these two streams need to engage so that smart strategies can be identified to improve both aims simultaneously using codependent, but distinct policy.
- Risk and human rights are two areas of convergence between UHC and GHS.
- Divergence appears in the conceptualisation of risk at the collective or individual level, and the prioritisation of domestic or global activity.

**What are the recommendations for policy and practice?**

- Health systems strengthening can be the policy mechanism which, brings GHS and UHC together, elevating health and mitigating risk for all.

## Introduction

Global health security (GHS) and universal health coverage (UHC) are frequently regarded as two sides of the same coin,^1^ or more cynically as a marriage of convenience.^2^ Yet, there has been little consideration of how these ideals interact, with academics and policymakers assuming that actions for one will also be advantageous to the other. This paper analyses at a macro level where these ideals converge, and where differences lie both conceptually and empirically. We argue both GHS and UHC focus on the mitigation of risk and human rights. Mitigating the risk of individuals who face impoverishment owing to healthcare expenditure is core to UHC. For GHS, the risk is transnational and emerges from outbreaks with cross-border potential. Hence, the bearer of the risk, and the appropriate steps to mitigate it, are different in each agenda and may sometimes be in conflict. Similarly, while both agendas enshrine human rights and we see convergence through the realisation of the right to health, we see distinctions between economic, cultural and social rights with civil and political rights.

It is important to address these differences before considering the mutual opportunities offered by their ‘marriage’, to ensure that inherent differences are not jettisoned for pragmatic reasons, risking distortion of local health priorities. We support the link that health system strengthening (HSS) creates opportunity to connect GHS and UHC in a tangible way, with clear policy pathways that can benefit both ideals.

### Defining Global Health Security (GHS) and Universal Health Coverage (UHC)

We recognise that the definition of UHC can vary in distinct, but convergent ways.^3^ A holistic definition is ensuring individuals have access, without discrimination to comprehensive, appropriate and timely, quality health services determined at the national level according to needs, as well as access to safe and affordable medicines, while ensuring that the use of these services do not expose users to financial difficulties.^4^ However, for the purpose of this paper, we focus within this definition on the extent to which the costs of healthcare are covered.^5^ We recognise that such a definition is not comprehensive, but we also acknowledge that the two components of UHC (access and risk protection) are in tension when it comes to decision making about provision, particularly in resource-poor settings, as the goal of access would lead to prioritisation of the most (cost) effective services, whereas a focus on financial protection would favour allocation of resources to more expensive interventions.^6^ However, in stressing the importance of universal access to effective healthcare, and universal financial protection against the costs of this care, the definition is consistent with the United Nations Sustainable Development Goals (SDGs), which includes in Goal 3 "ensure healthy lives and promote well-being for all at all ages" and in particular target (3.8) to “achieve UHC including financial risk protection, access to quality essential healthcare services and access to safe, effective, quality essential medicines and vaccines for all”.^7^

We define GHS activities as those concerned with preventing, detecting and responding to infectious disease threats of international concern to limit any socioeconomic impact of transborder disease, which mirrors the WHO definition.^8^ Nevertheless, we recognise that GHS is “very much like a chameleon” “essentially contested” and “not adequately defined”.^9–12^

GHS is underpinned by a legal instrument, the International Health Regulations (2005) (IHR).^13^ The IHR provide guidance for how states should develop and maintain their national capacities to minimise public health threats. While there is no binding international legal equivalent for UHC, the International Covenant on Economic, Social and Cultural Rights guarantees the human right to health. General Comment 14 on the right to health, which provides interpretive guidance on the right to health, proposes a framework of availability, accessibility, acceptability and quality.^14^ Moreover, policy initiatives create normative guidance on how to implement UHC, including The World Health Report 2010,^5^ Making Fair Choices on the Path to UHC and the United Nations General Assembly 67/81.^15^ Similarly, GHS has the policy and operational work of the Global Health Security Agenda (GHSA), an international partnership launched in 2014 and now comprising over 60 countries, international organisations and non-governmental stakeholders,^16^ which provides political impetus and international collaboration to meeting IHR requirements.

### Current intersections between the two concepts

Five key works have sought to connect GHS and UHC. Jain and Alam highlight that UHC can help advance GHS.^17^ First, low or no financial barriers to accessing healthcare stimulates demand for health services which facilitates early infectious disease detection. Second, protecting people from catastrophic financial risk reduces an individual’s possibility of falling into poverty, an important social determinant of infectious disease spread.^18^

Yates, Dhillon and Rannan-Eliya echo Jain and Alam’s first thesis verbalising “the availability of accessible and universal healthcare services in all countries is the crucial first line of defence for all against such threats to health”.^19^ Moreover, if people are unable to access healthcare in their local communities, this increases the likelihood of individuals crossing borders to seek healthcare, thus increasing the risk of onward transmission internationally.^19^ This work shows how these two concepts are mirrored empirically: suggesting that UHC’s relationship between financial protection and equitable distribution of risk (which addresses people’s ability to pay, while protecting the sick), mirrors the relationship between donor and recipient states for GHS, whereby wealthy states finance outbreak responses in affected states. While this risk pooling is not part of the IHR or GHSA mandate, it can be argued that IHR compliance should reflect the ability to pay while protecting weaker states.^19^

Moreover, Yates *et al* highlight that movements towards UHC build trust.^2 19^ This form of trust may exist between governments and populations, between health providers and patients and between financiers and recipients of health. This trust may foster effective collaboration when an outbreak emerges, improving public compliance with state-led interventions to limit disease spread.^20 21^ However, Ooms *et al* are more sceptical of joining the two agendas together, recognising that they are synergistic, but not self-evidently so.^2^ In resource-poor settings, they recognise distinct policy pathways for UHC and GHS; for example, whether to fund development of surveillance capabilities or social health insurance mechanisms, a point we would agree with.

Ooms *et al* further underscore the instrumentalism in linking these agendas. Tying UHC to GHS may provide greater leverage for financing UHC^2^ (Yamey echoes this suggestion, that while the world’s gaze is on GHS in the wake of Ebola, associating these can be a tool for getting attention to UHC and the health of populations in low and middle-income (LMIC) settings^22^). Conversely, GHS advocates may connect with the UHC agenda to gain legitimacy among those who conceive of the security discourse being too focused on high income country (HIC) interests.^2^ However, Ooms *et al* conflate UHC and HSS. These are used interchangeably, and this risks unclear understandings of what UHC entails, furthering the potential for misaligned priorities.

The fifth work considers GHS as “collective” security and “individual” security which broadly aligns with UHC.^23^ Heymann suggests that a difference exists between collective health security concerned with mutual global vulnerabilities posed by transborder spread of acute public health issues, and individual health security which includes access to safe and effective health services, products and technologies.^23^ Heymann’s argument follows that if there is individual health security, this contributes to collective health security at the community, national and global levels (i.e GHS).

### Conceptual convergence: risk

Both UHC and GHS aim to mitigate potential health and economic threats either at the level of the individual (UHC) or the collective (GHS). For UHC, one such risk results from individuals’ exposure to economic hazard as a result of a health event, that is, an individual’s health needs may be met only by incurring impoverishing or catastrophic costs associated with accessing appropriate healthcare.^19^ This form of individual or familial risk is centred on the cost, rather than the type of illness and can relate to acute to chronic conditions. Anyone may be exposed to this financial risk, the potential exposure is a lifetime, the likelihood of occurrence is high, and the consequences of exposure are disproportionately large for the poor who have insufficient funds to ensure financial resilience when confronted with a health concern.^24^ However, UHC offers an effective risk reduction intervention: proposing prepayment and pooling mechanisms to reduce both the probability of healthcare-related losses occurring, and the severity of their impacts on household’s budgets when they do. This also enhances individuals’ willingness and ability to access healthcare as opposed to delaying careseeking until they become very ill, thereby driving up healthcare costs for everyone. Accordingly, risk reduction through UHC benefits both individuals and societies. Moreover, reducing risk to any health concern through UHC, including communicable disease, has significant opportunity costs for GHS.

Instead of the ‘livelihood risk’ for UHC, the risk for GHS results from an infectious disease hazard which may result in a large-scale outbreak, threatening a population and/or economic or political stability as a result of opportunity costs lost through interrupted access to international markets, reduced international travel and fear among the population. Despite the IHR seeking to minimise such disruption, there are several examples of factors beyond a government’s control during an outbreak which impact a range of sectors beyond health.^25–27^ Indeed, President Ellen Johnson Sirleaf argued that the best action the USA could take to support Liberia in the Ebola epidemic was to “not ostracise us via trade”, suggesting that severing economic ties would pose as much risk as the virus itself (Emily Mendenhall, personal communication, 2017).

Accordingly, GHS focuses on future-proofing pandemic risk through preparedness. It does this by contingency planning for a range of disease threats.^28^ Luckily, large-scale international outbreaks are rare events, nevertheless, the severity of the potential (socio)economic impact of an outbreak leads to considerable investment in risk mitigation. This inadvertently may bias the public’s risk perception, creating potentially disruptive influences on “business as usual” for international travel and trade.^12 29^ Exemplifying this was the West-Africa Ebola epidemic, which had a relatively low likelihood of ‘anyone in the globe’ becoming infected, because of the low reproductive ratio of the disease. Nevertheless, despite the low actual risk, there was a high perceived risk. Margaret Chan reflected “I have never seen a health event strike such fear and terror, well beyond the affected communities”.^30^ This fear led to the implementation of expensive policies such as airport screening apparatus in HICs. These were not instrumental in reducing the actual risk of disease incursion but were effective political placebos implemented by governments to reduce perceived risks felt by HIC citizens.

### Conceptual convergence: human rights

Heymann’s distinction between GHS as collective security and UHC as individual security allows convergence between the two agendas through the lens of human rights also. Achieving both GHS and UHC require states to comply with their obligations and duties under international, regional and domestic human rights law. Human rights are often conceptualised as matters of individual security, whereby a state fails to respect, protect or fulfil an individual’s human rights. However, even where an individual successfully seeks recourse against a state for a human rights violation, such decisions have a collective impact, setting precedent that results in the state complying with its human rights obligations elsewhere. This is particularly the case for UHC, where human rights actions launched by individuals have, according to some proponents, addressed underlying systemic failures by governments to take steps to immediately realise the right to non-discrimination and progressively realise the right to health.^31^ These latter obligations typically fall within the realm of economic, social and cultural rights. This requires states to progressively realise these rights to the maximum of their available resources, while not regressing from steps already taken for non-discrimination and meeting minimum core obligations.^32^ In contrast, much of the dialogue discussing GHS and human rights relates to civil and political rights, such as those codified in the IHR; rights that the state must respect, provide and fulfil such as the rights to life, freedom of movement, and freedom from torture or cruel, inhuman or degrading treatment.^33^ While this civil and political rights framing is understandable as it focuses on the short-term and immediate vulnerability of individuals to the state’s actions when seeking to protect the many and/or the economy during an outbreak, the goals of GHS are fundamentally grounded in economic, social and cultural rights, namely, the right to health. The right to health includes the obligation that states take steps necessary for the “prevention, treatment and control epidemic, endemic, occupational and other diseases”.^34^ This obligation is congruent with GHS, and is also codified in the IHR, for example, within the core capacity obligations.

As a result, convergence between UHC and GHS can be found through the realisation the right to health, with both UHC and GHS requiring that states address inaction or regression in realising the right to health to the mutual benefit of both ideals.

### Conceptual divergence: inward versus outward: individual versus global security

Despite unifying features, there are differences in each with respect to the characterisation, who is identified as “at risk” and what responses have been taken to mitigate risk. We suggest these understandings of risk mirror divergent conceptualisations of security.

GHS has sought to answer two questions: security from what and for whom?^12^ We know that the ‘from what’ is different in the case of UHC and GHS, as outlined above, but so too is the ‘for whom’. For UHC, at risk is the everyday person who may be affected by ill health and the associated costs, or the inability to access health services due to other non-financial barriers. For GHS, however, the global population is at risk as their chances of contracting an infectious disease are reduced through ensuring GHS. Others have argued that the referent object for GHS is the economy or national security of a particular state fearing the socioeconomic impact of an outbreak on trade and travel.^35 36^ Accordingly, GHS predominantly mitigates risk from the top down, and UHC may mitigate risk from the bottom up, although infrastructure and support is required from the state to support individuals in risk pooling behaviour.

Both UHC and GHS risks are mitigated by financial investment in health. For UHC, the investment reduces the time people delay care-seeking due to the financial burden of paying for health. Through GHS, the investment is in pandemic preparedness; strengthening surveillance and response mechanisms to respond to infectious disease outbreaks under IHR (2005). Consequentially, the rationales and methods for mitigating against these risks—from the household to state levels—are quite different.

While private and non-profit actors are vital in global health, we argue that states play a fundamental role in the convergence of the two risks identified in this paper. However, a distinction emerges between mitigating a state’s risks which are domestic priorities, and those that are globally focused. For instance, state priorities that are domestically focused may involve prepayment schemes to reduce the financial risks posed to citizens (UHC). On the other hand, states prioritising GHS focus on implementing the IHR (2005) to reduce the risk of severe economic impact in the case of an acute public health event (Wenham, Examining Sovereignty in Global Health, PhD, 2016). These risks are fundamentally different, although the policies deployed may carry opportunity costs for both UHC and GHS goals. Governments, particularly in resource-constrained settings must decide whether to prioritise their global or domestic responsibilities, based on which risk they consider the most important. National leaders may prioritise one agenda over the other, aligning with political and economic priorities; for example, they may prioritise UHC when fighting an election as it is popular with the domestic electorate, yet focus on GHS when looking to attract donor dollars.

### Practical convergence: HSS

We argue that HSS can be the policy mechanism which brings GHS and UHC together, elevating health and mitigating risk for all. This echoes Kutzin and Sparkes who argue, “health system strengthening is what we do: UHC, health security and resilience is what we want”.^3^

A health system can be defined as the ensemble of all public and private organisations, institutions and resources involved in the improvement, maintenance or restoration of health.^37^ HSS refers to policy and programmatic activity designed to apply systems thinking to health, to improve overall performance.^38^ The WHO framework for HSS encapsulates six building blocks: service delivery, health workforce, health information systems, medical products, health financing and leadership and governance.^39^ The health system shapes many people’s health by determining how s/he accesses medical care, from whom s/he receives medical care, what medicines are available and accessible, what technologies are affordable and available for testing and diagnostics and how s/he is expected to pay for it,^39^ and as such contains many of the tenets of UHC.

For UHC, functioning health systems organised around people, institutions and resources leads to improved access, quality, sustainability and affordability for individuals.^38^ For GHS, successfully functioning health systems underpin countries’ ability to detect and respond to disease threats.^39^ In this way, a response to a health emergency (GHS) should be embedded within an existing health system, involving Farmer’s interweaving of “stuff, staff, systems and space” to address the needs of an epidemic and population health.^40^ Kluge expands this, providing suggestions for how to interlink these concepts, noting that investing in HSS improves GHS, so that systems become resilient to health crises and can respond when needed.^41^ By investing in health systems, this increases the resilience of states to respond to outbreaks of disease that spread across national borders, thereby investing indirectly in GHS.^3 38^ HSS therefore is a common road to both UHC and GHS.^3^

### Indicators convergence

**Figure 1:**
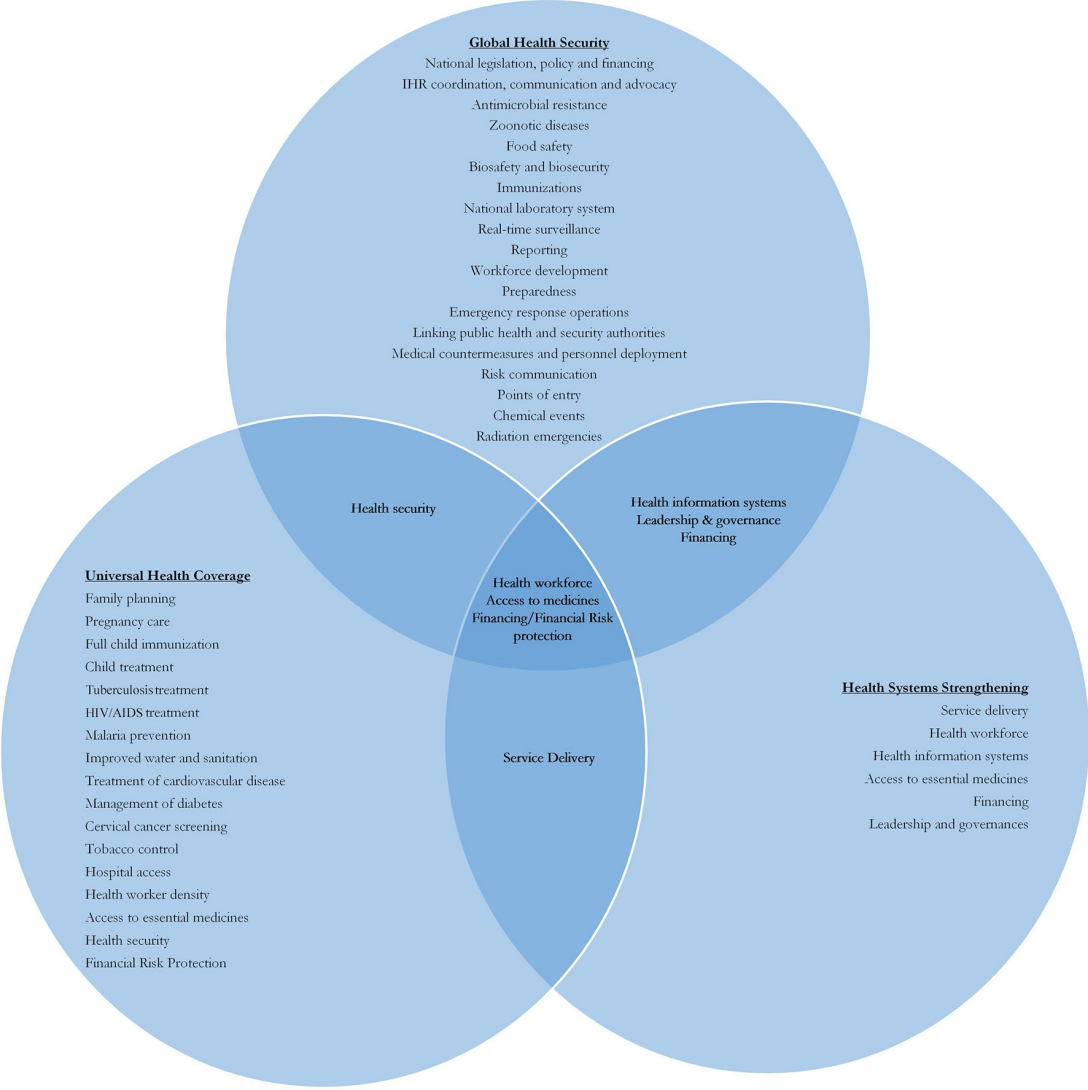
Synergy between global health security (GHS), universal health coverage (UHC) and health systems strengthening (HSS).

Beyond the conceptual, we assessed convergence and divergence of UHC, GHS and HSS based on policy metrics. As these concepts are embedded within key pieces of global policy, it seemed appropriate to use these indicators to ascertain whether there was practical as well as conceptual convergence between goals. We mapped GHS, using the first edition of the Joint External Evaluation Tool indicators as a proxy, and UHC, using SDG indicators 3.8.1 and 3.8.2, to measure health service coverage and financial protection^24 42 43^ and HSS, using the six WHO Building Blocks. As these indicators link to each policy aim, where we see convergence is a direct evaluation of how the concepts overlap. Figure 1 shows a tepid synergy between UHC and GHS. Although UHC indicators explicitly include reference to GHS, in a catchall “Health Security IHR Core Capacity Index”, it is not a key component of the index. Convergence appeared in financing, health workforce availability and capacity and access to medicines. There was not even overlap between the “infectious disease” indicators of UHC and those of GHS. However, despite limited overlap between GHS and UHC, there is considerable overlap between HSS and both GHS and UHC, with each of the six building blocks finding a comparable indicator with the other two agendas, and all three goals focusing on health workforce, access to medicines and financing/financial risk protection.

### Concerns linking these agendas

Synergising GHS and UHC raises several red flags. For UHC focusing on health through prepayment risks prioritising curative clinical services at the expense of individual and population health promotion and prevention.^44^ This leads to more healthcare services but worse outcomes overall and distributed benefits less equitably.^44^ For GHS, the limitation is its focus on national and economic security and the threat of infectious disease amid trade routes. This prioritises diseases which affect dominant trading networks of HICs, creating a quasi-postcolonial power imbalance denoting which diseases are considered globally important.

There remain health issues which fall outside of both of GHS and UHC (and HSS) priority setting. Recognising the conditions that are systematically excluded from both agendas is equally important. For example, road safety, improvement of Water, Sanitation and Hygiene (WASH) facilities, pest control and neglected disease which are core to improving human health, but neglected in both UHC and GHS.^44^ Yet, many donors expect discrete funding priorities that can be easily measured, such as treatment for the big three. Indeed, addressing the potential economic threats to national labour force through a multitude of further health burdens may be one way to align the concept of “risk” between UHC and GHS.

A further challenge is resource allocation: in healthcare systems worldwide, there are gaps between available funding and possible health interventions leading to priority setting.^3 15^ What are the ethical, political and socioeconomic implications of prioritising GHS, which may threaten HICs, as well as LMICS, rather than addressing Non-Communicable Diseases (NCDs) relating to the growing tobacco epidemic in Africa or ultraprocessed food in South America? Priority setting implies difficult choices have to be made and raises important ethical and equity considerations. UHC requires decision makers to agree on criteria and establish transparent and fair priority setting processes.^15^ Further elaboration is needed to understand how concerns for GHS and UHC can be considered within this.

Additionally, open definitions of “health systems” and how to measure their strength leave the door open for an emphasis on GHS and entire disease areas (such as NCDs) that does not address health inequities within a country with limited resources. Like Unicef’s support of selective primary healthcare in the 1980s—which was introduced as a means to simplify and actualise primary healthcare goals—and the Gavi and Global Fund approach to HSS,^45^ promoting an HSS model that includes both GHS and UHC means promoting those parts of a health system that overlap between the two frameworks and overlooks what falls outside. Accordingly, we must consider what defines a strong health system on an individual country basis that must address both the individual’s and the population’s needs.

## Conclusion

UHC and GHS are increasingly linked in global health policy. This paper illuminated the potential synergies between the two parallel agendas, but has considered the inherent tensions of a joined up UHC-GHS framework. We consider risk as being a unifying conceptual tool: the risk of the international spread of infectious disease on a population and national/economic security is fundamental to GHS. For UHC, the risk centres on the threat of financial impoverishment due to catastrophic health expenditures. However, these agendas are not comprehensively aligned. We recognise divergence between these frameworks; between the individual and the collective and between domestic and international priorities. Empirically, we show there are some overlapping indicators between GHS and UHC, but there are also a number of indicators outside this synergy. To that extent, the UHC and GHS goals are in tension. The research and practice communities that represent these two streams need to engage so that smart strategies can be identified to improve both aims simultaneously using codependent, but distinct policy. We suggest HSS as a method to achieve both and in doing so build more “equitable and sustained improvements across health services and health outcomes”.^39^ Yet, we caution that this is not panacea, but a meaningful step to bringing these global health agendas together in a more comprehensive mechanism.
